# Mid-Term Follow-Up Study of Children Undergoing Autologous Skin Transplantation for Burns

**DOI:** 10.3390/life13030762

**Published:** 2023-03-11

**Authors:** Angyalka Válik, Katalin Harangozó, András Garami, Zsolt Juhász, Gergő Józsa, Aba Lőrincz

**Affiliations:** 1 Department of Paediatrics, Medical School, University of Pécs, 7 József Attila Street, H-7623 Pécs, Hungary; 2 Division of Paediatric Surgery, Traumatology, Urology and Paediatric Otolaryngology, Department of Paediatrics, Medical School, University of Pécs, 7 József Attila Street, H-7623 Pécs, Hungary; 3 Department of Thermophysiology, Institute for Translational Medicine, Medical School, University of Pécs, 12 Szigeti Street, H-7623 Pécs, Hungary

**Keywords:** paediatric, burn, skin, graft, scar, POSAS, Vancouver Scar Scale

## Abstract

Deep partial and full-thickness burns require surgical treatment with autologous skin grafts after necrectomy, which is the generally accepted way to achieve permanent wound coverage. This study sought to examine the grafted and donor areas of children who underwent autologous skin transplantation, using two assessment scales to determine the severity of the scarring and the cosmetic outcome during long-term follow-up. At the Surgical Unit of the Department of Paediatrics of the University of Pécs, between 1 January 2015 and 31 December 2019, children who had been admitted consecutively and received autologous skin transplantation were analyzed. Twenty patients met the inclusion criteria in this retrospective cohort study. The authors assessed the results using the Patient and Observer Scar Assessment Scale (POSAS) and the Vancouver Scar Scale (VSS). There was a significant difference in how parents and examiners perceived the children’s scars. In the evaluation of the observer scale, the most critical variables for the area of skin grafted were relief and thickness. Besides color, relief was the worst clinical characteristic on the patient scale. However, when medical professionals evaluated the donor site, significantly better results were obtained compared to the transplanted area (average observer scale score: 1.4 and 2.35, *p* = 0.001; VSS: 0.85 vs. 2.60, *p* < 0.001), yet it was similar to the graft site in the parents’ opinion (Patient Scale: 2.95 and 4.45, *p* = 0.181).

## 1. Introduction

A burn injury is classified as a trauma caused by direct or indirect thermal effects. Depending on the source, combustions can be etiologically categorized as scalding, contact, electrical, flame, radiation, chemical, or friction [1]. According to European data, burns mainly affect children under five years of age and are predominantly caused by scalding. The hands are most often affected, followed by chest and facial injuries, leading to complications in functionally and aesthetically crucial areas [2].

The severity depends primarily on the depth, extent, and localization. Secondarily, it is influenced by underlying medical conditions and aggravating factors. Such factors are open flame or electrical burns, inhalation injuries, age under five years, burns affecting more than 30% of the body surface, electrical trauma, and delayed or inadequate treatment [3].

Thermal injuries are most commonly classified based on their depth, because this corresponds best with the severity and, thus, the patient’s treatment. According to the categorization issued by the European Burns Association (EBA), the following groups can be distinguished: superficial (I), which only injures the epithelium and resolves spontaneously. Superficial partial-thickness (II/A), where burns damage the papillary layer yet are still capable of re-epithelialization. On the other hand, deep partial-thickness (II/B) combustions injure the reticular stratum of the skin and require surgical attention. Finally, full-thickness (III) thermal injuries destroy the subcutis or even deeper tissues [4]. Another important criterion for classifying burns is the extent of the combustion injury, expressed as a percentage of the total burned surface area (TBSA%) [5]. These categories are usually determined during physical examination, although it is possible to measure them objectively, which will be discussed briefly later.

After administering anesthesia, analgesia, and fluid resuscitation, the wounds must be cleaned and disinfected. Debridement may be performed mechanically by abrasion, ultrasound, or water jet. Probably the closest to an ideal wound-cleaning method is enzymatic degradation (for example, utilizing bromelain), which explicitly targets necrotic tissues. Deep partial- and full-thickness combustions require surgical treatment with necrectomy and autologous skin transplantation, which is a widely accepted way to achieve permanent coverage [6,7]. Split-thickness skin grafts are harvested from the donor site using a dermatome during transplantation. Due to the partially intact dermis layer, the affected area is capable of spontaneous epithelialization [8]. Therefore, its treatment is equivalent to managing superficial partial-thickness (II/A) burns. Based on this, positive results and minimal scarring at the donor site are expected. Alternatively, 3D-printed skin substitutes and tissue cultures from the patient’s own cells may be used for wound closure. They can seal the injury with minimal donor site involvement; however, these methods are costly, culturing takes weeks to complete, and if the cells are from a different organism, there may be a severe immunological reaction.

Temporary coverage of the donor and graft sites is necessary during healing, to provide a moist microenvironment, decrease fluid loss, and form a barrier against pathogens. Various dressings, from silver-containing ointments and biosynthetic porcine xenografts to burn hydroregulation-specialized, triple-layered hydrofiber bandages, are available. Additionally, amnion membrane allografts and tilapia xenografts are prevalent, while some developing countries apply banana skins and potato peels. However, their optimal utilization, regarding different demographic groups and types of injuries, and their relative effectivity are still debated [9,10]. Nevertheless, interventions with the fewest dressing changes should be preferred, to reduce the discomfort inflicted on the child. Consequently, this diminishes the narcotic doses required for pain management during bandage reapplications.

After the initial hospital treatment, the primary goal of rehabilitation is to prevent infection and rejection of the graft [11,12]. It is also essential to ensure the best possible range of motion and functional status, thus preventing the development of contractures. Compression garments and physiotherapy play a significant role in this [13,14]. Scars that form during the healing of thermal injuries often become hypertrophic, and their management is problematic. Risk factors for abnormal wound healing include darker skin tone, female gender, and young age. Neck or upper limb combustions, repeated surgical procedures, mesh graft use, or higher burn severity also increase the hazard of a graver scar outcome [15,16]. Scar extent can be quantified using different scar assessment scales. However, there are numerous methods, and their results are hard to translate to other classifications [17,18]. The Patient and Observer Scar Assessment Scale (POSAS) [19,20] and Vancouver Scar Scale (VSS) [21] are scoring systems developed for better transparency and more accurate follow-up.

Early compression treatment is vital for avoiding scar hypertrophy. Compression garments can be used after desquamation has stopped [13]. This method reduces blood flow and oxygenation to the affected area, creating an imbalance between the collagen synthesis and breakdown, resulting in a flat scar. In our experience, conservative treatment may affect the scar quality for 1–1.5 years after the injury. Wounds that cause contractures and limit mobility should be treated surgically, without delay. This is especially true in infants, due to the possibility of growth deformations. In turn, this would require an additional complex reconstruction, which might lead to inferior outcomes.

The aesthetic outcomes of scars can be adequately assessed during an extended follow-up. Regrettably, data on these endpoints are rarely reported in the literature [18,19]. Therefore, we aimed to describe children’s transplanted and donor site scar characteristics, years after the injury. We chose the two scores mentioned previously to examine the scar quality from the guardians’ and medical professionals’ perspectives and to evaluate these scales’ interchangeability.

## 2. Materials and Methods

### 2.1. Design

We conducted a nonrandomized, single-center, mid-term follow-up clinical trial at the Surgical Division, Department of Paediatrics, Medical School, University of Pécs, Pécs, Hungary. Children’s demographic data were collected retrospectively, between 1 January 2015 and 31 December 2019. The Institutional Review Board of Paediatric Surgery provided ethical approval in 2020. Control appointments took place throughout 2021.

During the follow-up, first, we took photographic documentation from the operated areas and then measured the children’s POSAS 2.0 [22] and VSS [23] scales via physical examination. Measurements were not performed by the physician alone but included the head nurse of the pediatric surgical department (K.H.), to increase objectivity. Subsequently, the patient’s guardians received information explaining the investigation and signed an informed consent. Finally, the parents evaluated their child’s operated skin.

It must be mentioned that, although we observed and rated the aspects within the scales separately for the donor site during the study, we only considered the overall opinion and the total score in our evaluation.

### 2.2. Participants

Children ((1) younger than 18 years old) who (2) visited our clinic between 1 January 2015 and 31 December 2019, (3) with deep partial or full-thickness burns, and (4) treated with autologous split-thickness skin transplantation were eligible for this study. Inclusion criteria also encompassed (5) ≤10% TBSA burn surface and (6) localization in the trunk or limb regions. In addition, (7) attending the mid-term (more than three years after the injury) follow-up and (8) completed questionnaires were required to participate in this study. Regrettably, we excluded 24 patients from our study due to missing photo documentation and absent follow-up investigations.

Exclusion criteria included children with (1) comorbidities, (2) those receiving full-thickness skin grafts, or (3) facially localized injury, due to their distinctive regenerative properties [24,25,26]. In conclusion, 20 children with deep thermal injuries requiring autologous skin transplantation were included in this study.

### 2.3. Assessment

#### 2.3.1. POSAS-Patient Scale

Two numerical scales are contained within the POSAS. One is completed by the medical examiner and the other by the patient (or guardian in the case of children), based on their observations and impressions [19,27]. In this case, the critical difference was that parents were asked guided questions about the characteristics.

The first part is the patient scale, which contains seven questions (Figure S1).

Six of these questions are related to the patient’s (in this case, the parent’s) assessment of the scar’s characteristics, such as pain, itching, color, stiffness, thickness, or irregularity. During the analysis, the clinical features included in the scale are divided into sensory and physical characteristics. These questions adequately cover the well-established features of scar formation that are considered necessary in the clinical evaluation of a burn scar. The seventh and final question concerns the overall general opinion of the patient.

Each question was rated by the guardian on a scale of 1 to 10, where one indicates no difference between a scar and healthy skin and where 10 is the worst outcome or sensation imaginable for the child. Combustions are often extensive and can have a high degree of heterogeneity within a scar [28]. For this reason, we asked the parents to assess the scar or scars as a whole, rather than to pick out a single detail.

#### 2.3.2. POSAS-Observer Scale

**Figure 1:**
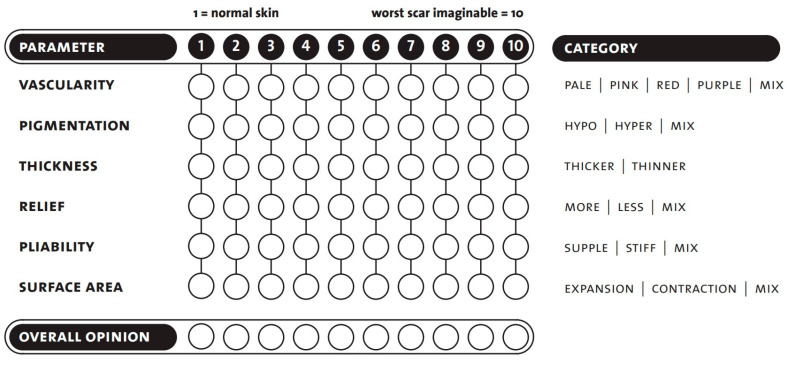
POSAS-observer scale template. Seven questions are rated, in the range of 1 to 10. Copyright © Prof. Dr. Paul van Zuijlen. All rights reserved [22,29].

The observer scale contains six parameters to be tested and is completed with an overall opinion. As with the patient scale, the scoring is between 1 and 10, with a score of one indicating that the scar area is equivalent to healthy skin and 10 being the worst imaginable scar (Figure 1) [19,22].

When assessing vascularity, we had to ensure that there were functional blood vessels in the scar tissue [30]. Increased vascularization of the scar causes color changes (pink, red, purple). Applying pressure to the scar with a finger (some examiners used the help of transparent plexiglass) could therefore eliminate the color change caused by the circulation. Vascularity is a good indicator of scar activity in the early phase. After a particular time, this activity decreases, and the redness partially disappears.

The next group was pigmentation disorders (hypo- and hyperpigmentation). They are caused by changes in the concentration of melanocytes and their melanin production. Unfortunately, significant variations are likely to remain long-term.

Another category was the thickness, which is the average distance between the subcutaneous-dermal border and the epidermal surface of the scar. The scar tissue will usually be broader than the surrounding skin in the first few months. Following this period, the scar thickness typically decreases. Some scars may remain hypertrophic (red, thick, and often itchy), while others may be atrophic (thin, pale, and vulnerable). Softening of the scar was assessed concerning the adjacent healthy skin surfaces.

Pliability as a skin quality was also assessed. It was tested by holding the wound between the index and thumb fingers and squeezing it. Scar tissue is generally less flexible than average skin, due to its thickness, stiffness, contraction, and adhesion. This can cause functional damage, primarily if they are located on or around joints.

The last measured parameter was the surface area of the scar, which is given relative to the original wound area. Surfaces may decrease or increase as the wound contracts or expands. Scar contraction is usually considered a complication while treating burn scars, as it can lead to functional problems, by reducing the range of motion.

#### 2.3.3. VSS

The most commonly used scar assessment scale is VSS, which measures four parameters [21,23]. These include vascularity, pigmentation, pliability, and height (Table 1). It goes from 0 to the maximum score of 13, corresponding to the worst rating. These factors were evaluated solely by medical professionals. Although we observed and marked the aspects within the scale separately during the study, only the overall opinion (total score) was considered in this evaluation.

### 2.4. Statistical Analysis

As only paired categorical values were investigated in our study, a Chi^2^-test was conducted using Monte Carlo simulation using an R-based algorithm (R Foundation for Statistical Computing, Vienna, Austria). The significance level was defined at *p* < 0.05. Means, standard deviations, and ranges were evaluated in Excel 2021 (Microsoft Corporation, Redmond, WA, USA). Comparisons between scoring systems were calculated after normalization (scaling the VSS values between 1–10), but the aforementioned descriptive statistics are reported in their original form.

## 3. Results

Twenty children’s demographic data and scar scores were analyzed in our study. The patients’ average age was 4.95 years, 70% were below five, and 30% were younger than 1 (SD: 5.33, range: 1–18). An equal sex distribution was observed, with an average burnt area of 5.35% TBSA (SD: 3.04, range: 1–9). Six patients (30%) had full-thickness injuries; the rest was deep partial only. Scalding was the dominant etiology, affecting 60% of patients, followed by contact (30%) and flame (10%) burns. Chest injuries were the most frequently affected location (50%), followed by hand (30%) and lower limb combustions. The average follow-up time of the patients was 4.35 years (SD: 1.24, range: 3–6 years).

Examiners used scales to evaluate the different clinical characteristics of the transplanted and donor areas in advance; we first describe the parent’s perceptions.

### 3.1. Transplanted Area

#### 3.1.1. POSAS-Patient Scale’s Results

Sensory parameters included pain and itching. Regarding pain (mean: 1.10, SD: 0.30), 90% of patients responded with a score of 1 and the rest with 2 (range: 1–2). Similarly, there were excellent results for itching (average: 1.20, SD: 0.87), where 95% of the parents rated it identical to regular skin, but one child scored a surprisingly high 5 (range: 1–5).

**Figure 2:**
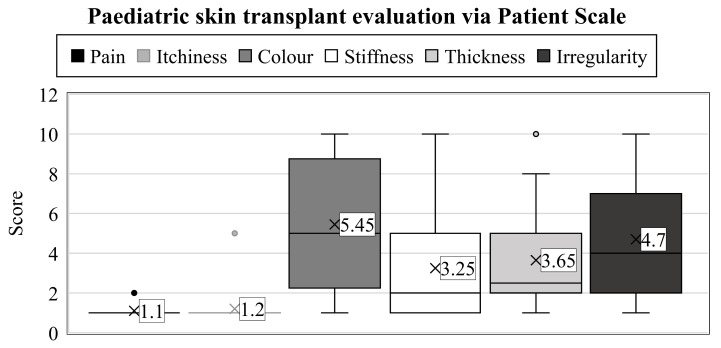
Years after thermal injuries, the guardians used the patient scales to assess their child’s transplanted skin between 1 and 10. The numbers represent the groups’ average value. Interquartile ranges are shown by the boxes’ height, with a median line separating them into rectangles. Maximum values can be examined at the top of the vertical lines (i.e., whiskers), and minimums at the lower end. Dots denote the outliers.

When characterizing the physical qualities, a wide variety of results were observed (range: 1–10) (Figure 2).

First, the parents had to assess the color of the scar (which we described as pigmentation and vascularity on the observer scale). In contrast to the results obtained on the observer scale, the outcomes were notably inferior here (mean: 5.45, SD: 3.07). Only one parent thought the result was the same as a healthy skin surface; in 20% of cases, it was evaluated as a 2, 15% of parents assessed it as a 5, while 20% responded with a score of 10, which is the worst possible outcome on the scale.

More favorable scores were recorded in the case of scar stiffness (average: 3.25, SD: 2.51), where 30% of children scored equivalent to uninjured skin, and another 25% rated them closely similar (2 points). Furthermore, two scores of 5 and only one of 10 were recorded, which was a better result than in the color assessment.

Opinions on thickness were, on average, between the two previous parameters. Twenty per cent of parents rated the scar’s width as 1 and 30% as 2. Therefore, these scores did not indicate any difference in this parameter in half of the cases compared to healthy skin areas. In addition, 2–2 parents rated the current condition as 5 and 10.

The last clinical feature to be assessed was the irregularity of the scar. As with the color test, only one child scored 1, and 25% of cases were a 2. Three children received a 5, and 10% were rated as 10.

Similarly to the observer scale, the POSAS patient scale was completed by asking for an overall opinion, leading to an average grade of 4.45 (SD: 3.15, range: 1–10). A statistical synopsis of the guardians’ evaluation regarding the transplant zone is shown in Table 2.

#### 3.1.2. POSAS-Observer Scale’s Outcomes

**Figure 3:**
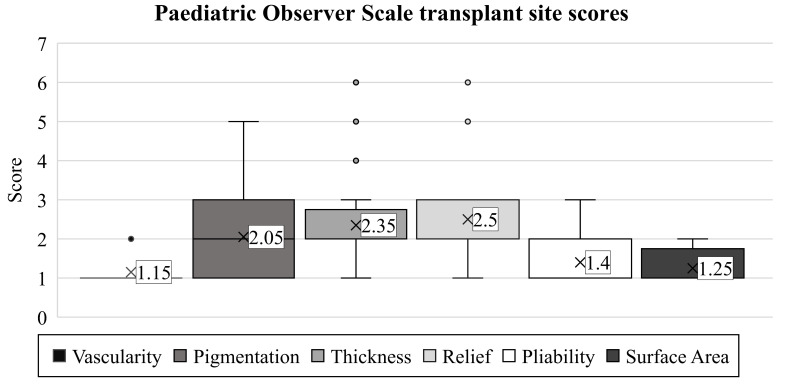
Based on the POSAS-observer scale, the transplanted skin qualities of children with burn injuries were evaluated. Notations are the same as detailed in Figure 2.

The scar’s vascularity was studied initially with the observer scale (mean: 1.15). Later, this proved to be the ideal observed endpoint, rated equivalent to the vascularization of healthy skin in 85% of children (Figure 3). Only three patients had different scores, with minimal deviations (range: 1–2, SD: 0.36).

We found a more notable difference among the children when assessing pigmentation (average: 2.05, range: 1–5, SD: 1.12). However, we observed results similar to healthy skin areas in only 40% of patients. The worst evaluation was a score of 5, present in one child.

Similarly, as in the case of pigmentation, the scores for thickness varied in the children (mean: 2.35, range: 1–6, SD: 1.28). The thickness of the scar matched the surrounding skin in only 20% of the patients. The majority (55%) received a score of 2, representing only a minimal discrepancy, while one patient had an exceptionally poor rating of 6.

**Figure 4:**
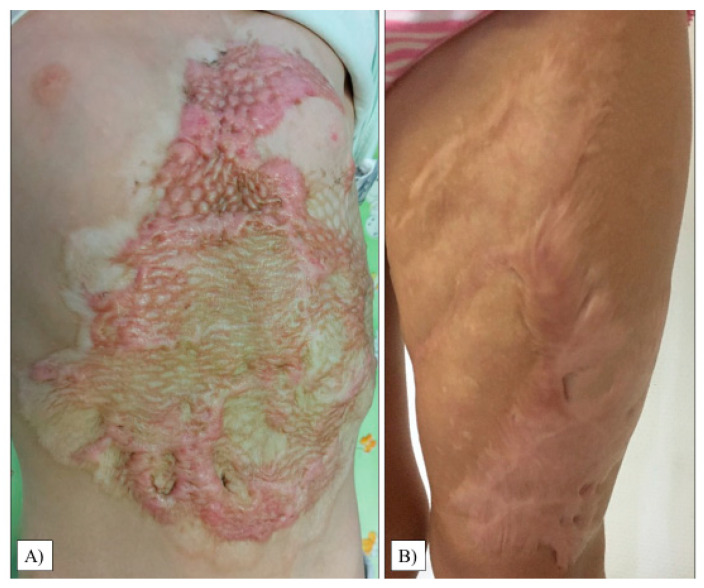
Hypertrophic scar formation in the transplanted chest and abdomen areas (**A**). Another patient had extensive scarring on her left thigh (**B**).

The scar relief was the most critical parameter in our study (average: 2.50, range: 1–6, SD: 1.36). As with the thickness, we observed only 20% of the children with a condition equivalent to healthy areas. Images of the most excessive scars in the transplant sites are shown in Figure 4.

Pliability showed the third-best outcome (mean: 1.4, SD: 0.66, range: 1–3). In most children (55%), the scar had the same pliability as normal skin on the rest of the body. Four children were given a score of 2, and two a score of 3.

Besides vascularity, the surface area of the scar was found to be the least problematic variable (mean: 1.25, SD: 0.43, range: 1–2). For this endpoint, 75% of the examiners observed results equivalent to healthy skin.

After characterizing the six parameters for describing each child’s scar, the examiners concluded with a general opinion on the scar using the POSAS observer scale. The average score was 2.35. Here, completely healthy outcomes (score of 1) were found in only 40% of the cases (SD: 1.53, range: 1–6).

#### 3.1.3. VSS Results

The mean score of the patients was 2.60. Utilizing VSS, twenty per cent of children had a near-perfect score, while 35% were rated a 2. The worst score was 7, observed in just one child (SD: 1.80, range: 0–7). We discovered that the results were similar to the POSAS observer scale (*p* = 0.7734), yet they differed significantly from the patient scale scores (*p* = 0.0023).

A statistical summary of the medical examiner’s view is shown in Table 3.

### 3.2. Donor Site

#### 3.2.1. POSAS Patient Scale Outcomes

**Figure 5:**
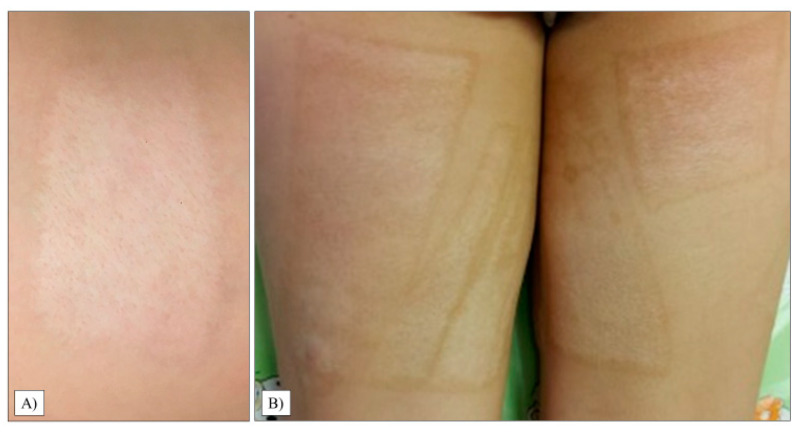
Donor areas on the thigh’s proximal posterior (**A**) and anteromedial part (**B**).

Although it was not statistically significant (*p* = 0.2508), there was a considerable difference between the parents’ and medical examiners’ perceptions, as guardians generally rated the quality of the children’s donor area as worse. The patients’ average grade was 2.95, with the majority of the guardians presenting a score of 1 and 2. In one case, the worst result reached the maximum rating of 10 (SD: 3.15, range: 1–10), which can be observed in Figure 5B.

#### 3.2.2. POSAS Observer Scale Results

When inspected by professionals, the study’s results confirmed our hypothesis that donor sites would receive lower scores on average (*p* = 0.0012). In 75% of the children, we found an area almost identical to healthy skin. The mean score was 1.4 (SD: 1.52, range: 1–4). The child who received the worst mark on the patient scale also had the poorest outcome here; however, it was significantly lower, only a 4 (Figure 5B).

#### 3.2.3. VSS Results

**Figure 6:**
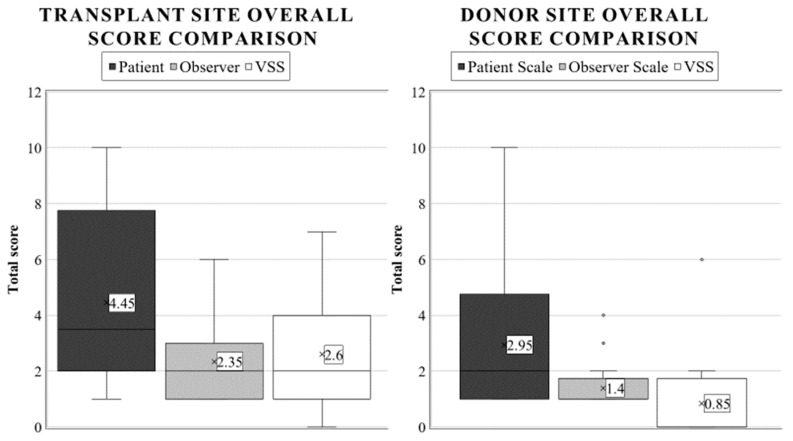
Average overall score appraisal using different burn scar scales in pediatric transplant and donor areas. The annotations are as described in Figure 2.

The results obtained from the VSS were very similar to the POSAS observer scale (*p* = 0.7734) and very distinct from the patient scale average. There was a significant difference between the donor and graft sites’ VSS ratings (*p* < 0.0001), further validating our hypothesis that the zone of burn injury will be more severely affected. In this case, the mean outcome of the patients was 0.85 (SD: 1.80, range: 0–6). Overall (as with the observer scale), 75% of the results were completely satisfying. Additionally, we graded the donor area of 20% of patients as 2, and one child was rated with an exceptionally high score of 6. Comparative boxplots about the total donor site scores using different scales are presented in Figure 6.

## 4. Discussion

Children after a burn are at compounded risk of morbidity, especially scarring, as their skin and immune and nervous systems are still developing. Therefore, a similar thermal trauma will cause a more profound and larger surface area skin injury compared to in an adult. However, in comparable TBSA% and depth injuries, they are likely to regenerate more rapidly after non-ionizing combustions, because their cell division is faster [31,32].

When evaluating these scars, more severe cases naturally received higher scores in this study. Due to the various dressings applied to the operated areas, and the relatively small sample size, we did not try to measure the effectiveness of the applied interventions. Our most important observation was that during scar assessment, there was a significant difference between the patient’s (in our case, the parent’s mean overall score) and the medical professional’s perception of the grafted area (Patient Scale: 4.45, Observer Scale: 2.35, *p* = 0.0155). Parents generally had a more negative opinion of their children’s outcomes. This view was likely due to the medical professionals’ experience with severe cases; thus, they tended to underestimate the individual burden of the condition on the patient. Another hypothesis is that due to the parents’ closer relationship with their child, they saw more vividly the problems originating from these scars. Of course, the full answer most probably contains both explanations and other variables as well.

Interestingly, this difference was not so evident when inspecting the donor sites; guardians and professionals judged them as statistically equal (Patient: 2.95 vs. Observer Scale: 1.4, *p* = 0.2508), although parents gave scores that were more than twice as high on average. Consequently, when deciding if further surgical reconstruction of a child’s skin is necessary, it is vital to average the patient’s and guardians’ views, because the medical personnel’s judgement does not entirely correlate with the child’s inconvenience.

When assessing the transplanted area, color (mean: 5.45) and irregularity (average: 4.7) were the most critical variables in the patient scale, with the highest scores. For the observer scale, the most challenging clinical characteristics were relief (mean: 2.5) and thickness (average: 2.35). Future research should concentrate on developing or optimizing interventions to preserve these skin qualities.

The healing response of the donor site, due to the partially preserved dermis layer, corresponds to a grade II/A lesion. Therefore, it was assumed that a more aesthetic result and higher scores would be obtained when evaluating the mean overall donor scores compared to the graft site. Our study only confirmed this hypothesis when weighing the assessment of medical examiners, where we found the most significant differences between these two areas (Observer Scale: 1.4 and 2.35, *p* = 0.0012; VSS: 0.85 vs. 2.60, *p* < 0.0001). In contrast, parents rated donor sites similarly to the transplant site with the patient scales (2.95 and 4.45, *p* = 0.1812).

Since scar assessment tools are numerous and ever-evolving, it is demanding to keep up-to-date with all of them and to be able to interpret the results quickly [17,18]. Even during the conduction of this trial, a novel POSAS version 3.0 was released. Therefore, another critical observation was that the results from the two scar evaluating surveys seem comparable after normalization, as shown by the equivalent overall outcomes when graded by professionals (*p* = 0.9966 in the case of the transplanted area and *p* = 0.7734 of the donor site). Given that another study found similar results in the facial region after a malignoma in adult patients, we can further verify the generalizability of these scales [33].

It is essential to mention that there are objective ways to describe a scar, such as thickness estimation with ultrasound or photogrammetry, which can also calculate burnt surface areas and depth. Likewise, color may be determined with reflectance spectroscopy or laser imaging. In addition, pliability, elasticity, and stiffness can be measured with a variety of methods, including tonometry and reviscometry. Artificial intelligence algorithms can further augment the precision and speed of these techniques [34].

However, the procedures mentioned above can be time-consuming, especially if there is a waiting list or transportation is needed for the hardware. Furthermore, the devices require expensive maintenance, software upgrades, calibration, and, most importantly, specialized staff to operate them and interpret their results. Moreover, there are still factors, such as pain and itching, which cannot be reported entirely objectively and unbiasedly, to our knowledge. Therefore, a swift severity estimation during a physical examination, which requires no equipment, will always be helpful.

The limitations of this analysis must also be reviewed. First, only a modest-sized population was analyzed, due to the long follow-up time required for scar formation. Initially, parents had problems interpreting some of the variables. We tried to counter this problem by explaining and describing them appropriately, to classify them as accurately as possible. It was also difficult for them to disassociate their evaluation, because most parents did not want to criticize the operating surgeon’s work; however, it was necessary to express their opinion. It is noteworthy that parents rated both the donor and the transplant sites similarly using patient scales (2.95 and 4.45, *p* = 0.1812), which suggests that some guardians may have potentially reported increased scar severity values (i.e., aggravation.) Overall, scar assessment scales are considered a subjective scoring system, because they are primarily based on the individual opinion of the examiner.

## 5. Conclusions

Hypertrophic scarring is a common complication of burn injuries, even after the best primary management. Therefore, it is essential to conduct extended follow-ups with children after deep combustions, to monitor their irregularities and provide interventions when needed. In addition, future research should focus on improving the scar’s color, irregularity, and thickness, as these were the worst endpoints after split-thickness skin transplantation.

When considering the severity of the scar, patients’ guardians had a significantly more negative opinion than the medical professionals; thus, this must be considered during surgical planning. Furthermore, the variety of scar scoring systems makes choosing the ideal assessment tool challenging. Consequently, noticing that the patient observer and Vancouver scales had similar results can help in interchanging and interpreting their outcomes.

## Figures and Tables

**Table 1:** The VSS scar assessment scale, grading between 0 and 13.

Score	Vascularity	Pliability	Height	Pigmentation
0	Normal	Normal	Flat	Normal
1	Pink	Supple	<2 mm	Hypopigmentation
2	Red	Yielding	2–4 mm	Hyperpigmentation
3	Purple	Firm	4 mm<	Mixed pigmentation
4		Banding		

**Table 2:** Summary of the patient scale scoring results.

	POSAS-Patient Scale-Transplant Site						
	Pain	Itchiness	Colour	Stiffness	Thickness	Irregularity	Overall Opinion
mean	1.1	1.2	5.45	3.25	3.65	4.7	4.45
SD	0.30	0.87	3.07	2.51	2.78	2.70	3.15
min	1	1	1	1	1	1	1
max	2	5	10	10	10	10	10

**Table 3:** Transplanted skin characteristics according to the medical examiners’ opinion.

	POSAS-Observer Scale-Transplant Site							VSS
	Vascularity	Pigmentation	Thickness	Relief	Pliability	Surface Area	Overall Opinion	
mean	1.15	2.05	2.35	2.5	1.4	1.25	2.35	2.6
SD	0.36	1.12	1.28	1.36	0.66	0.43	1.53	1.80
min	1	1	1	1	1	1	1	0
max	2	5	6	6	3	2	6	7

## Data Availability

Data are contained within the article.

## Supplementary Materials

The following supporting information can be downloaded at: https://www.mdpi.com/article/10.3390/life13030762/s1, Figure S1: Patient (or guardian) scar assessment with POSAS 2.0. Copyright © Prof. Dr. Paul van Zeitlin. All rights reserved [22].
