# Supplementary figures and images for: Mid-Term Follow-Up Study of Children Undergoing Autologous Skin Transplantation for Burns
Source: Life (Basel). 2023 Mar 11;13(3):762. doi: 10.3390/life13030762 (PMC10053383; doi:10.3390/life13030762)

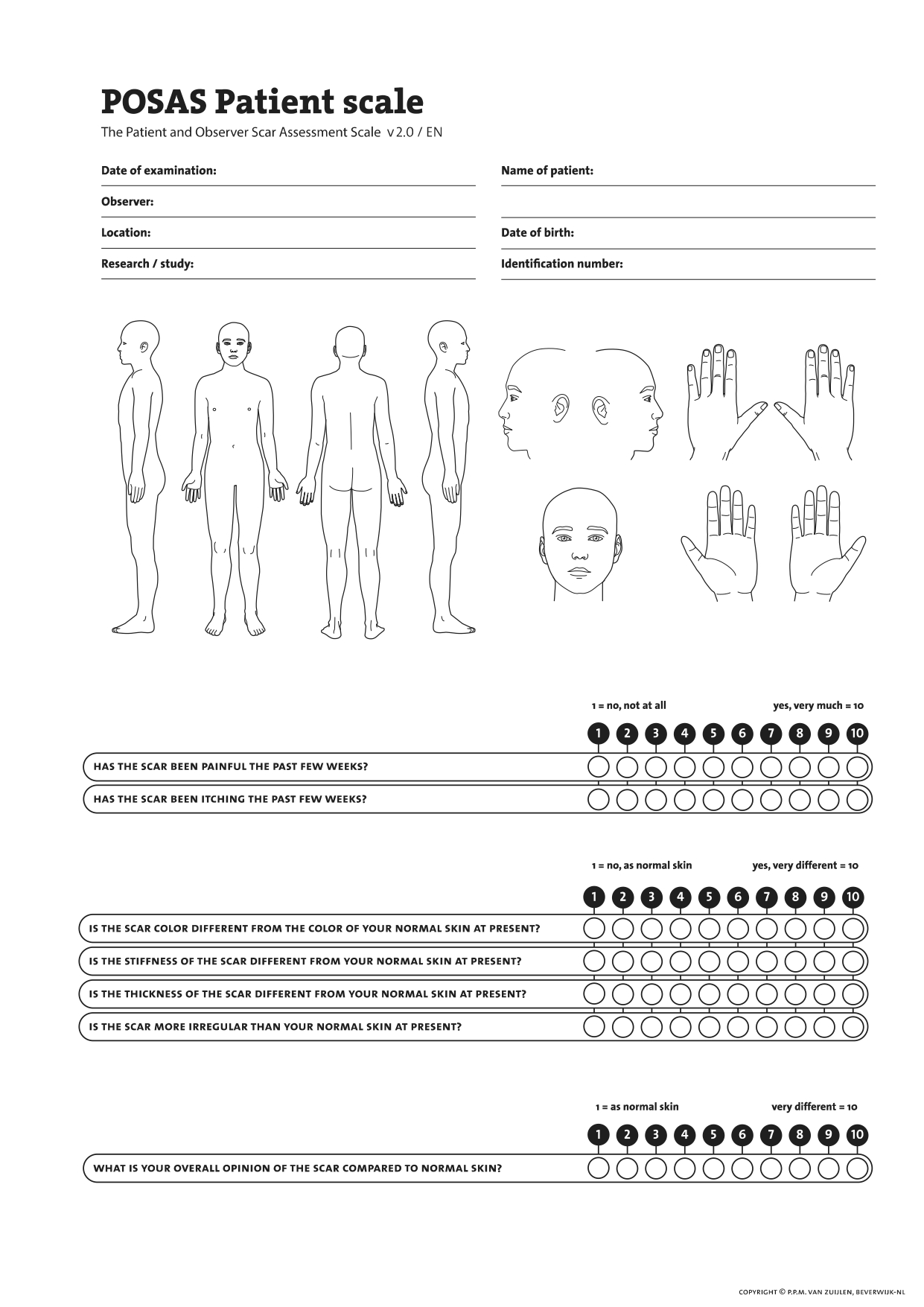

Supplement: Supplementary file 1 [file life-13-00762-s001.zip › life-2128242-supplementary.jpg]
